# Production of Albuminous Urine by Cantharides

**Published:** 1847-09

**Authors:** 


*Production of Albuminous Urine by Cantharides.*—In a communication on Cystitis from cantharides, made by M. Lavalleé to the Academy of Medicine, at its sitting on the 15th inst., it is stated by the author that under the use of this vesicatory, at whatever part of the body it may be applied, and however distant from the hypogastric region, albumen is liable to show itself in the urinary apparatus, under three forms: 1, in solution: 2, as a deposite in the urine: and 3, as a false membrane produced in the bladder. The albumen is contained in the urine under these circumstances in much greater proportion than in Bright's disease:—the author states that in some instances, after a day's rest, the deposite constitutes more than half the height of the liquid. As an exception, the urine may contain no traces: but then vesical symptoms, such as pain and tenesmus, are absent. This is a new form of artificial albuminuria."—*Ibid, from L'Union Médicale.*

